# Supplementary material for: Effect of Sowing Date and Environment on Phenology, Growth and Yield of Lentil (Lens culinaris Medikus.) Genotypes
Source: Plants (Basel). 2023 Jan 19;12(3):474. doi: 10.3390/plants12030474 (PMC9922022; doi:10.3390/plants12030474)
Supplement: Supplementary file 1 [file plants-12-00474-s001.zip › plants-1902215-supplementary.pdf]

## Trangie Agricultural Research Centre climate 2018 (a)

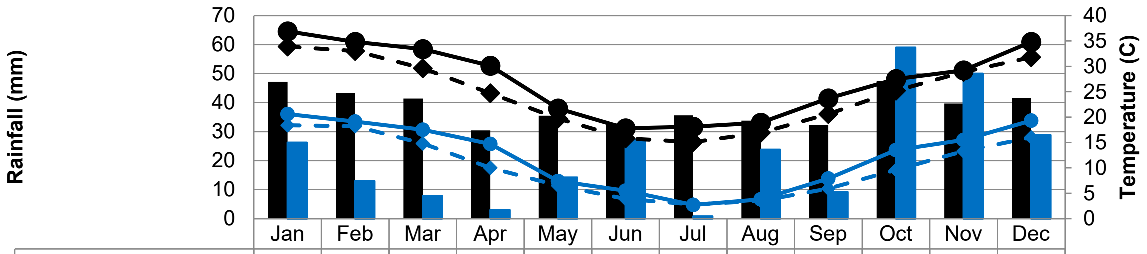


| | Jan | Feb | Mar | Apr | May | Jun | Jul | Aug | Sep | Oct | Nov | Dec |
|---|---|---|---|---|---|---|---|---|---|---|---|---|
| LTA rainfall | 47.2 | 43.4 | 41.4 | 30.4 | 35.5 | 32.8 | 35.6 | 33.8 | 32.3 | 47.5 | 39.7 | 41.5 |
| 2018 rainfall | 26.2 | 13.0 | 7.8 | 3.0 | 14.2 | 27.2 | 0.8 | 23.8 | 9.2 | 59.0 | 50.0 | 28.8 |
| LTA min temp | 18.4 | 18.2 | 14.8 | 10.0 | 6.5 | 3.9 | 2.8 | 3.5 | 5.9 | 9.6 | 13.3 | 16.0 |
| 2018 min temp | 20.6 | 19.1 | 17.5 | 14.7 | 7.4 | 5.5 | 2.7 | 3.8 | 7.9 | 13.5 | 15.5 | 19.3 |
| LTA max temp | 33.9 | 33.0 | 29.6 | 24.7 | 19.7 | 15.8 | 15.1 | 16.9 | 20.6 | 25.2 | 28.9 | 31.8 |
| 2018 max temp | 36.9 | 34.8 | 33.4 | 30.1 | 21.7 | 17.8 | 18.1 | 18.9 | 23.7 | 27.5 | 29.2 | 34.8 |

## Trangie Agricultural Research Centre climate 2019 (b)

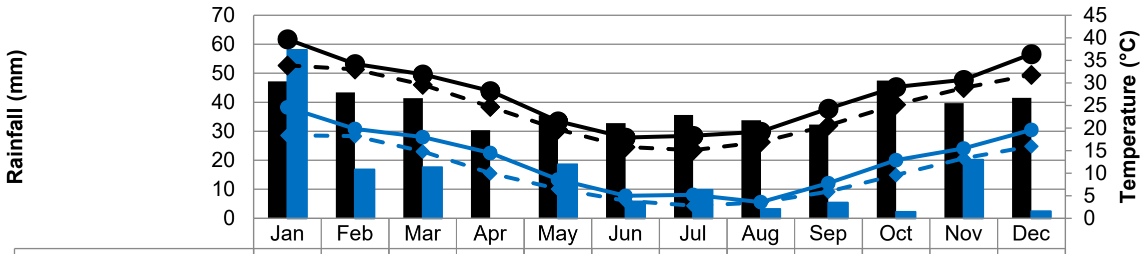


| | Jan | Feb | Mar | Apr | May | Jun | Jul | Aug | Sep | Oct | Nov | Dec |
|---|---|---|---|---|---|---|---|---|---|---|---|---|
| LTA rainfall | 47.2 | 43.4 | 41.4 | 30.4 | 35.5 | 32.8 | 35.6 | 33.8 | 32.3 | 47.5 | 39.7 | 41.5 |
| 2019 rainfall | 58.0 | 16.8 | 17.6 | 0.0 | 18.6 | 5.8 | 9.8 | 3.2 | 5.4 | 2.2 | 20.2 | 2.4 |
| LTA min temp | 18.4 | 18.2 | 14.8 | 10.0 | 6.5 | 3.9 | 2.8 | 3.5 | 5.9 | 9.6 | 13.3 | 16.0 |
| 2019 min temp | 24.6 | 19.8 | 18.0 | 14.5 | 8.6 | 5.0 | 5.2 | 3.6 | 7.8 | 12.9 | 15.5 | 19.6 |
| LTA max temp | 33.9 | 33.0 | 29.6 | 24.7 | 19.7 | 15.8 | 15.1 | 16.9 | 20.6 | 25.2 | 28.9 | 31.8 |
| 2019 max temp | 39.7 | 34.2 | 31.9 | 28.2 | 21.5 | 17.9 | 18.3 | 19.2 | 24.3 | 29.1 | 30.7 | 36.4 |

## Wagga Wagga Agricultural Institute climate 2018 (c)

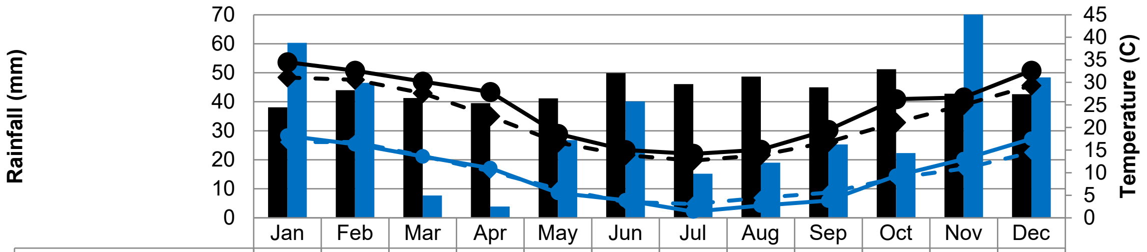


| | Jan | Feb | Mar | Apr | May | Jun | Jul | Aug | Sep | Oct | Nov | Dec |
|---|---|---|---|---|---|---|---|---|---|---|---|---|
| LTA rainfall | 38.1 | 44.0 | 41.3 | 39.5 | 41.2 | 49.9 | 46.1 | 48.7 | 45.0 | 51.2 | 42.8 | 42.6 |
| 2018 rainfall | 60.3 | 46.5 | 7.7 | 3.9 | 26.8 | 40.1 | 15.2 | 19.0 | 25.3 | 22.3 | 91.3 | 48.4 |
| LTA min temp | 16.8 | 16.8 | 13.7 | 10.3 | 6.4 | 3.5 | 3.1 | 4.4 | 5.6 | 8.8 | 10.9 | 14.5 |
| 2018 min temp | 18.2 | 16.3 | 13.6 | 11.0 | 5.5 | 3.9 | 1.4 | 2.7 | 3.8 | 9.3 | 13.0 | 17.5 |
| LTA max temp | 31.1 | 30.6 | 27.6 | 22.5 | 16.7 | 13.9 | 12.7 | 13.9 | 16.8 | 21.1 | 25.0 | 29.3 |
| 2018 max temp | 34.5 | 32.6 | 30.2 | 27.9 | 18.6 | 15.0 | 14.2 | 15.1 | 19.5 | 26.3 | 26.7 | 32.6 |

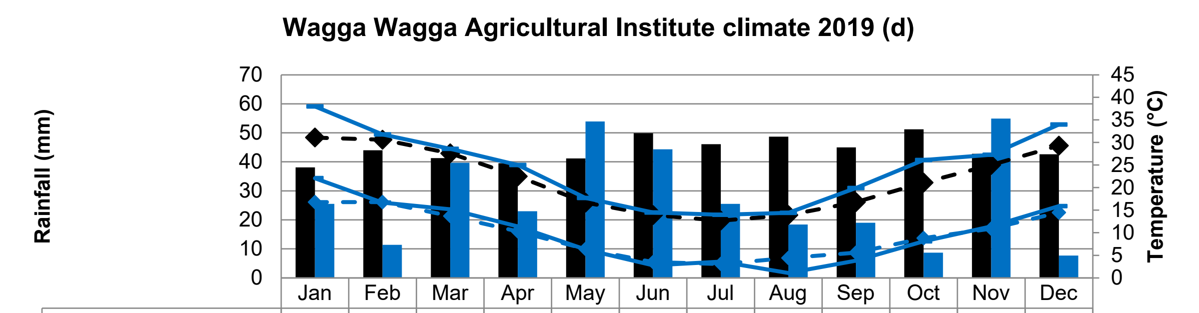


| | Jan | Feb | Mar | Apr | May | Jun | Jul | Aug | Sep | Oct | Nov | Dec |
|---|---|---|---|---|---|---|---|---|---|---|---|---|
| LTA rainfall | 38.1 | 44.0 | 41.3 | 39.5 | 41.2 | 49.9 | 46.1 | 48.7 | 45.0 | 51.2 | 42.8 | 42.6 |
| 2019 rainfall | 25.5 | 11.4 | 39.7 | 23.0 | 53.9 | 44.3 | 25.5 | 18.4 | 19.0 | 8.7 | 54.9 | 7.7 |
| LTA min temp | 16.8 | 16.8 | 13.7 | 10.3 | 6.4 | 3.5 | 3.1 | 4.4 | 5.6 | 8.8 | 10.9 | 14.5 |
| 2019 min temp | 22.1 | 16.7 | 15.2 | 11.4 | 6.3 | 2.8 | 3.6 | 1.1 | 3.8 | 8.1 | 11.4 | 15.9 |
| LTA max temp | 31.1 | 30.6 | 27.6 | 22.5 | 16.7 | 13.9 | 12.7 | 13.9 | 16.8 | 21.1 | 25.0 | 29.3 |
| 2019 max temp | 38.0 | 31.8 | 28.6 | 25.0 | 17.7 | 14.5 | 14.0 | 14.4 | 19.9 | 26.1 | 27.3 | 34.0 |

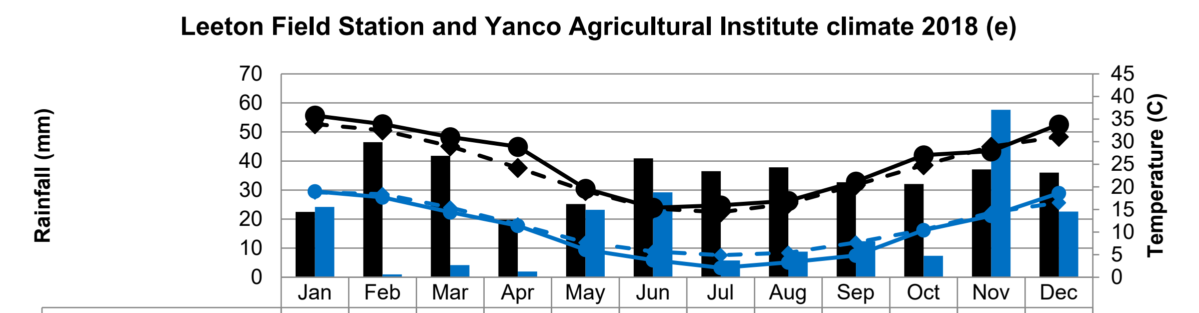


| | Jan | Feb | Mar | Apr | May | Jun | Jul | Aug | Sep | Oct | Nov | Dec |
|---|---|---|---|---|---|---|---|---|---|---|---|---|
| LTA rainfall | 22.5 | 46.5 | 41.8 | 19.8 | 25.2 | 40.9 | 36.5 | 37.8 | 32.7 | 32.1 | 37.1 | 36.0 |
| 2018 rainfall | 24.2 | 1.0 | 4.2 | 2.0 | 23.2 | 29.2 | 5.8 | 8.8 | 12.4 | 7.4 | 57.6 | 22.6 |
| LTA min temp | 18.8 | 18.4 | 15.4 | 11.7 | 7.7 | 5.7 | 4.9 | 5.4 | 7.7 | 10.5 | 14.3 | 16.5 |
| 2018 min temp | 19.0 | 17.7 | 14.4 | 11.4 | 6.1 | 3.8 | 2.1 | 3.3 | 4.8 | 10.4 | 13.6 | 18.6 |
| LTA max temp | 33.9 | 32.5 | 29.0 | 24.2 | 19.1 | 15.2 | 14.4 | 16.3 | 20.4 | 24.8 | 28.9 | 31.1 |
| 2018 max temp | 35.8 | 33.9 | 31.0 | 28.9 | 19.6 | 15.4 | 15.9 | 16.9 | 21.2 | 27.0 | 27.9 | 33.8 |

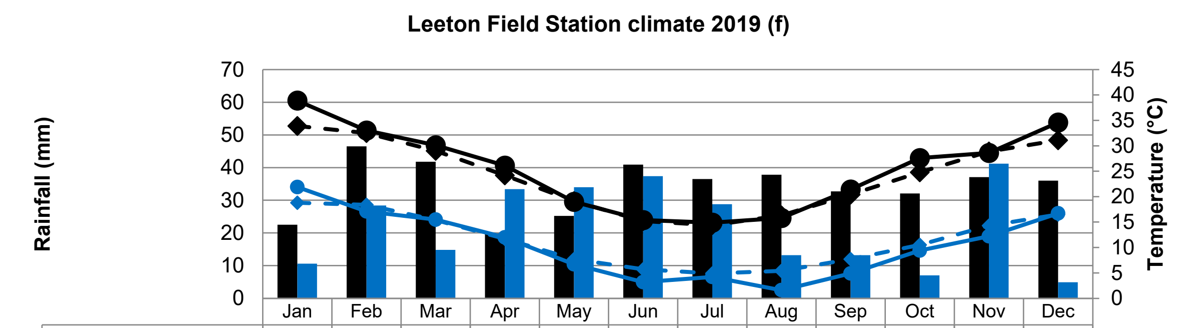


| | Jan | Feb | Mar | Apr | May | Jun | Jul | Aug | Sep | Oct | Nov | Dec |
|---|---|---|---|---|---|---|---|---|---|---|---|---|
| LTA rainfall | 22.5 | 46.5 | 41.8 | 19.8 | 25.2 | 40.9 | 36.5 | 37.8 | 32.7 | 32.1 | 37.1 | 36.0 |
| 2019 rainfall | 10.6 | 28.4 | 14.8 | 33.4 | 34.0 | 37.4 | 28.8 | 13.2 | 13.2 | 7.0 | 41.2 | 4.9 |
| LTA min temp | 18.8 | 18.4 | 15.4 | 11.7 | 7.7 | 5.7 | 4.9 | 5.4 | 7.7 | 10.5 | 14.3 | 16.5 |
| 2019 min temp | 21.9 | 17.1 | 15.5 | 12.0 | 6.7 | 3.2 | 4.2 | 1.6 | 4.9 | 9.4 | 12.2 | 16.7 |
| LTA max temp | 33.9 | 32.5 | 29.0 | 24.2 | 19.1 | 15.2 | 14.4 | 16.3 | 20.4 | 24.8 | 28.9 | 31.1 |
| 2019 max temp | 38.9 | 33.0 | 30.1 | 26.1 | 19.0 | 15.4 | 14.9 | 15.8 | 21.4 | 27.6 | 28.6 | 34.6 |

**Figure S1.** (**a**–**f**) Rainfall, minimum (min) and maximum (max) temperatures recorded in 2018 and 2019, together with the long-term averages (LTA) at TARC, WWAI, LFS and YAI.
